# Systematic Review of Gut Microbiota and Major Depression

**DOI:** 10.3389/fpsyt.2019.00034

**Published:** 2019-02-11

**Authors:** Stephanie G. Cheung, Ariel R. Goldenthal, Anne-Catrin Uhlemann, J. John Mann, Jeffrey M. Miller, M. Elizabeth Sublette

**Affiliations:** ^1^Division of Consultation-Liaison Psychiatry, Columbia University, New York, NY, United States; ^2^Department of Psychiatry, Columbia University, New York, NY, United States; ^3^Molecular Imaging & Neuropathology Area, New York State Psychiatric Institute, New York, NY, United States; ^4^Division of Infectious Diseases, Department of Medicine, Columbia University, New York, NY, United States; ^5^Microbiome & Pathogen Genomics Core, Columbia University, New York, NY, United States; ^6^Department of Radiology, Columbia University, New York, NY, United States

**Keywords:** depression, gut, microbiome, microbiota, gut-brain axis

## Abstract

**Background:** Recently discovered relationships between the gastrointestinal microbiome and the brain have implications for psychiatric disorders, including major depressive disorder (MDD). Bacterial transplantation from MDD patients to rodents produces depression-like behaviors. In humans, case-control studies have examined the gut microbiome in healthy and affected individuals. We systematically reviewed existing studies comparing gut microbial composition in MDD and healthy volunteers.

**Methods:** A PubMed literature search combined the terms “depression,” “depressive disorder,” “stool,” “fecal,” “gut,” and “microbiome” to identify human case-control studies that investigated relationships between MDD and microbiota quantified from stool. We evaluated the resulting studies, focusing on bacterial taxa that were different between MDD and healthy controls.

**Results:** Six eligible studies were found in which 50 taxa exhibited differences (*p* < 0.05) between patients with MDD and controls. Patient characteristics and methodologies varied widely between studies. Five phyla—*Bacteroidetes, Firmicutes, Actinobacteria, Fusobacteria*, and *Protobacteria*—were represented; however, divergent results occurred across studies for all phyla. The largest number of differentiating taxa were within phylum *Firmicutes*, in which nine families and 12 genera differentiated the diagnostic groups. The majority of these families and genera were found to be statistically different between the two groups in two identified studies. Family *Lachnospiraceae* differentiated the diagnostic groups in four studies (with an even split in directionality). Across all five phyla, nine genera were higher in MDD (*Anaerostipes, Blautia, Clostridium, Klebsiella, Lachnospiraceae incertae sedis, Parabacteroides, Parasutterella, Phascolarctobacterium*, and *Streptococcus*), six were lower (*Bifidobacterium, Dialister, Escherichia/Shigella, Faecalibacterium*, and *Ruminococcus*), and six were divergent (*Alistipes, Bacteroides, Megamonas, Oscillibacter, Prevotella*, and *Roseburia*). We highlight mechanisms and products of bacterial metabolism as they may relate to the etiology of depression.

**Conclusions:** No consensus has emerged from existing human studies of depression and gut microbiome concerning which bacterial taxa are most relevant to depression. This may in part be due to differences in study design. Given that bacterial functions are conserved across taxonomic groups, we propose that studying microbial functioning may be more productive than a purely taxonomic approach to understanding the gut microbiome in depression.

## Introduction

Major Depressive Disorder (MDD) is a debilitating psychiatric illness affecting an estimated 300 million people worldwide (1). MDD is the leading cause of disability globally (2) and is associated with ~800,000 suicide deaths annually (3). Despite significant advances in our understanding of the etiology of MDD (4), existing knowledge is incomplete, treatments are inadequate, and new insights into MDD pathophysiology are urgently needed. One novel area of investigation related to MDD pathophysiology is the gut microbiome.

The microbiome is a collection of trillions of microorganisms, including bacteria, that inhabit and interact with human hosts, with effects ranging from beneficial to pathogenic (5), and also more specifically refers to the collection of microbiota and their genetic material (6). Groups of bacteria are organized on a phylogenetic tree with taxonomical categories ranging from low resolution (kingdom, phylum) to high resolution (genus, species) taxa.

The gut microbiota are considered so necessary and so integrated into host function that some describe this population as an overlooked organ (7). Beyond the breakdown of otherwise indigestible food substances and production of micronutrients, gut microbiota affect the hypothalamic-pituitary-adrenal axis (HPA) (8), produce neurologically active substances such as gamma-aminobutyric acid (GABA) (9) and short-chain fatty acids (SCFAs) (10), and influence the immune system and gut barrier (11–16).

A growing body of literature supports and characterizes a gut-brain axis, and elucidates a possible role of gut microbiome dysfunction in major depression. Associations between the gut microbiome and depression have been identified in studies of inflammatory states and gut barrier health (17–19). In addition, animal studies have supported the possibility of a causative role of dysbiosis (disruption of the microbiome) in depression-like behaviors. Broad-spectrum antibiotic administration in mice leads to dysbiosis, depression-like behavior, and altered neuronal hippocampal firing, with reversal of this phenotype following probiotic treatment with *Lactobacillus casei DG* (20). Male germ-free mice also exhibit elevated levels of serotonin (5-hydroxytryptamine) and its metabolite, 5-hydroxyindoleacetic acid in the hippocampus (21). Few translational studies are as dramatic as the human-to-rodent studies in which transplantation of gut microbiota from humans with MDD to germ-free or microbiota-deficient rodents induces a depression-like phenotype, including anhedonia and anxiety-like behaviors, not observed in mice receiving microbiota transplantation from healthy controls (22, 23). A small number of human studies have demonstrated behavioral correlates of dysbiosis ranging from infant colic (24) to behavioral abnormalities in neurodevelopmental disorders (25). Finally, several studies have compared the gut microbiome in affected vs. control individuals. In order to investigate the role of the gut microbiome in MDD, we systematically reviewed the scientific literature of case-control studies concerning gut microbiome composition in MDD.

## Methods

### Literature Search for Gut Microbiome Studies in MDD

To identify putative depression-relevant aspects of gut microbiome composition, a PubMed literature search was performed that included articles published prior to February 28, 2018 with combinations of the terms “depression,” “depressive disorder,” “stool,” “fecal,” “gut,” and “microbiome.” Additional relevant articles were sought through manual bibliography search. Eligibility criteria were: (1) articles in English; (2) human case-control studies; (3) articles focused on depression; and (4) gut microbiota quantified from stool samples. Two raters (SGC and ARG) reviewed all search results and came to a consensus about inclusion/exclusion of each article.

## Results

### Literature Search to Identify Human Case-Controlled Studies

**Figure 1:**
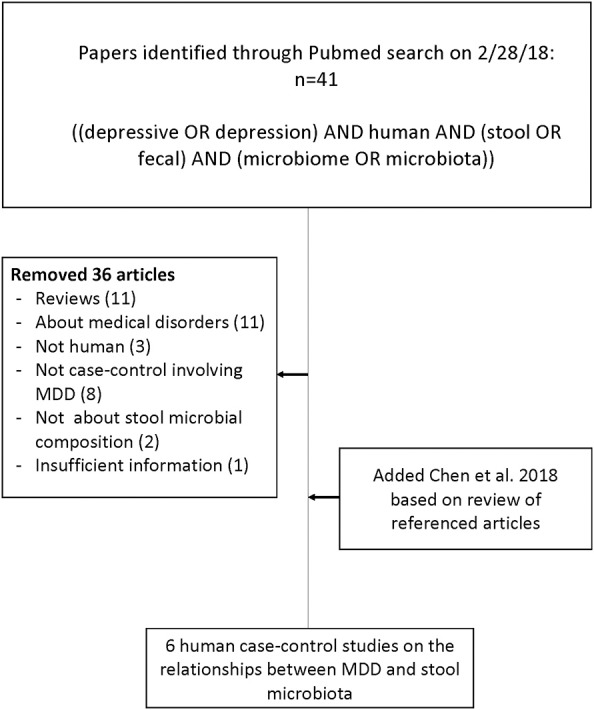
Flow chart of literature search for articles concerning case-control studies of the gut microbiome in major depressive disorder compared with healthy volunteers.

Six studies met the search criteria (23, 26–30), involving a total of 392 research participants, 204 MDD and 188 controls (see Figure 1).

### Characteristics of the Studies

#### Sample (see Tables 1, 2 for More Details)

Five of the six identified case-control studies were conducted in Asia: four in China and one in Japan. The sixth study was conducted in Norway. The sample sizes of the studies were relatively small, ranging from 10 to 63 individuals per diagnostic group. Three of the studies documented smoking status, but the number of active smokers was not matched between diagnostic groups.

**Table 1:** Population characteristics.

**Study**	**Country**	**Sample size**		**% Female**		**Age (Mean** **±*****SD*****)**		**BMI (Mean** **±*****SD*****)**		**% Smoking**		**Mean baseline depression severity**
		**MDD**	**Control**	**MDD**	**Control**	**MDD**	**Control**	**MDD**	**Control**	**MDD**	**Control**	**(MDD)**
Naseribafrouei et al. (26)	Norway	37	18	54.1	61.1	49.2 ± 13.9	46.1 ± 13.9	25.9 ± 4.2	24.7 ± 3.3	N/A	N/A	MADRS 26.3 ± 7.6
Jiang et al. (27)	China	A-MDD 29 R-MDD 17	30	A-MDD 37.9 R-MDD 47.0	50.0	A-MDD 25.3 ± 5.4 R-MDD 27.1 ± 5.4	26.8 ± 5.4	A-MDD 20.3 ± 3.4 R-MDD 21.8 ± 3.4	19.6 ± 3.4	A-MDD 10% R-MDD 12%	7%	A-MDD^*^ HAM-24 29.8 ± 7.6 MADRS 27.4 ± 8.5
Aizawa et al. (28)	Japan	43	57	41.9	61.4	39.4 ± 10.0	42.8 ± 12.7	23.2 ± 3.6	22.3 ± 3.7	N/A	N/A	HAM-21 16.9 ± 6.8
Zheng et al. (23)	China	58	63	62.1	63.5	40.6 ± 11.7	41.8 ± 12.3	22.0 ± 2.4	22.6 ± 2.5	18%	26%	HAM-17 22.8 ± 4.4
Lin et al. (29)	China	10	10	40.0	40.0	36.2 ± 10.1	38.1 ± 2.9	23.8 ± 1.9	24.2 ± 2.0	40%	30%	HAM-17 ≥ 23
Chen et al. (30)	China	10	10	50.0	50.0	43.9 ± 13.8	39.6 ± 9.0	23.5 ± 2.0	22.6 ± 1.5	0%	0%	HAM 25.6 ± 4.7

A-MDD, active-major depressive disorder; R-MDD, recovering-MDD who exhibited 50% reduction in HAM score after treatment; BMI, body-mass index; HAM, Hamilton depression rating scale; MADRS, Montgomery-Åsberg depression rating scale.

* *Baseline mean depression severity for R-MDD not provided*.

**Table 2:** Methodologic characteristics of the articles reviewed.

**Study**	**Genetic analysis**	**Diversity assessments**		**MDD**			**Pooled population**		
		**α- Diversity**	**β-Diversity**	**% on AD; AD treatment**	**Diagnostic/severity determination**	**Depression severity inclusion criteria**	**Medical history**	**Antibiotic usage**	**Probiotic, prebiotic, synbiotic usage**
Naseribafrouei et al. (26)	Illumina deep sequencing of 16S gene amplicons Region: unspecified Pipeline analysis: QIIME Database: RDB Cut-off: 1% Closed-reference picking	Species richness Simpson's D		73% on medications	Research Criteria of the ICD-10/MADRS	None	Included control participants with diffuse neurological symptoms, but who were not found to have cerebral disorders after neurological examination, CT, and MRI. One control participant (6%) was on antidepressant medication.	N/A	N/A
Jiang et al. (27)	454 pyrosequencing of the 16S rRNA gene Region: V1–V3 Pipeline analysis: Mothur Database: RDB Cut-off: 3% Closed- or open-reference picking unspecified	Shannon Simpson Evenness ACE Chao1	Principal Coordinate Analysis of Unweighted UniFrac distances	A-MDD: 72% on SSRIs or SNRI, 24% on atypical antipsychotic, 83% on benzodiazepine R-MDD: 100% on SSRI or SNRI, 29% on atypical antipsychotic, 58.9% on benzodiazepine	MINI used as screening for preexisting psychiatric disorders, SCID DSM IV / HAM-24, MADRS	HAM-17 score ≥ 20	Excluded individuals with hypertension, cardiovascular disease, diabetes mellitus, obesity, liver cirrhosis, fatty liver disease, irritable bowel syndrome, inflammatory bowel disease, known active bacterial, fungal or viral infections. Excluded MDD participants with other psychiatric disorders, drug, or alcohol abuse in the last year. Excluded control participants with psychiatric or physical illnesses.	No use of antibiotics month prior to fecal sample	No prebiotic or synbiotic use in the month prior to fecal sample
Aizawa et al. (28)	Yakult Intestinal Flora-SCAN® bacterial 16 S rRNA-targeted RT-qPCR			65%; average imipramine equivalent dose = 187.7 ± 152.7 mg/day	Interviewed using MINI and unstructured interview, DSM IV/HAM-21	None	Excluded individuals with history of central nervous system disease or any severe medical condition, substance abuse or dependence, mental retardation, history of surgery on the GI system, or severe congenital abnormality. Excluded control participants with current or past history of psychiatric illness or contact with psychiatric services. 48.3% of MDD group had IBS and 14% of control group had IBS using the Rome III Criteria.	No recent antibiotic use	5 individuals used probiotics
Zheng et al. (23)	16S rRNA gene sequencing using Illumina HiSeq2500 Region: V3–V5 Pipeline analysis: Mothur Database: RDB Cut-off: 3% Closed- or open-reference picking unspecified	Observed species Shannon Phylogenetic diversity Simpson	Weighted Bray-Curtis similarity Principal Coordinate Analysis of Unweighted UniFrac distances	33%; paroxetine (3), duloxetine venlafaxine, citalopram (3), mirtazapine, sertraline, fluoxetine, and ”others“ (8)	SCID DSM IV TR/HAM-17	None	Excluded MDD candidates who had a history of substance abuse, were pregnant, nursing or currently menstruating. Control participants were excluded if they had evidence of systemic medical illness, mental disorder or family history of psychiatric disorder.	Not currently taking	Not currently taking
Lin et al. (29)	16S rRNA gene sequencing using Illumina MiSeq sequences Region: V3-V4 Pipeline analysis: Mothur Database: Silva v.119 Cut-off: 3% Closed- or open-reference picking unspecified	ACE Chao Shannon Simpson	Principal Coordinate Analysis of weighted UniFrac distances	100%; escitalopram 10 mg/day	DSM-IV TR/HAM-17	HAM-17 ≥ 23	Included control participants with good physical and mental health, no stomach or gut problems.	None within last month	None within last month
Chen et al. (30)	Metaproteomics: phylogenetic analysis of the bacterial peptides			40%; 20% on SSRI or SNRI, 20% on atypical antipsychotics	SCID DSM IV / HAM (version not specified)	HAM > 20	Excluded participants who had pre-existing physical or other mental illnesses, history of drug or alcohol abuse, family history of any psychiatric disorder, infectious disease, gastrointestinal diseases, irritable bowel syndrome, inflammatory bowel disease, diabetes, obesity, smoking, and excluded females who were pregnant, nursing or menstruating.	Not currently taking	Not currently taking

*AD, anti-depressant; HAM, Hamilton depression rating scale (17, 21, or 24 item); ICD, international statistical classification of diseases and related health problems; MADRS, Montgomery-Åsberg depression rating scale; MINI, mini-international neuropsychiatric interview; SCID, structured clinical interview for DSM disorders; SSRI, selective serotonin reuptake inhibitor; SNRI, serotonin-norepinephrine reuptake inhibitor*.

##### Medication and use of probiotics/prebiotics/synbiotics

All studies included participants taking psychiatric medications. The percentage of individuals in the MDD group on antidepressant medication ranged from 33 to 100% among the six studies. While the majority of studies included individuals on a variety of antidepressant medications, Lin et al. (29) standardized the treatment to 10 mg of escitalopram per day. Of note, two studies (27, 30) included individuals being treated with antipsychotics. All but one study (26) excluded individuals on antibiotics within at least the last month. Three studies specifically excluded recent probiotic use (27, 29, 30), and one of these studies also excluded recent prebiotic or synbiotic use (27); however, five participants in one study (28) were on probiotics.

##### Psychiatric and medical conditions

Exclusion criteria related to psychiatric and medical conditions differed among the six studies. All but one (26) excluded a variety of specific medical illnesses or stipulated no active disease. Zheng et al. (23), problematically, excluded medical illness only in the controls. Some of the studies specified exclusion of individuals with other psychiatric history. For example, Aizawa et al. (28) excluded those with a history of substance use disorder and intellectual disability. Naseribafrouei et al. (26), did not recruit healthy volunteers but instead recruited controls with diffuse neurological symptoms who were not diagnosed with any neurological condition after physical examination and imaging. One study (27) recruited only MDD participants with scores >20 on the 24-item HAM at baseline but then divided the sample into responders and non-responders after 4 weeks of treatment with respect to microbiome findings.

#### Methodology (see Table 2)

##### Clinical assessments

Diagnostic methods included the International Classification of Disease 10 (ICD 10), the Mini-International Neuropsychiatric Interview (MINI), and the Structural Clinical Interview for DSM-IV (SCID). Depression severity was assessed variously by the Montgomery-Åsberg Depression Rating Scale (MADRS) and the Hamilton Depression Rating Scale (HAM). The majority of studies used the HAM (17, 21, and 24-item scales) to determine severity, whereas one study (26) used the ICD 10 and MADRS for severity measurements. Mean depression levels in all six studies were of moderate to high severity (see Table 1).

##### Microbiome quantification

All but one of the studies included used 16S rRNA gene sequencing, but with different region specification: region V1–V3 (27), region V3–V5 (23), region V3–V4 (29), no specified region (26), and a bacterial rRNA-targeted reverse transcription-quantitative polymerase chain reaction (RT-qPCR) method with no specified region (28). For pipeline analysis, one study used Quantitative Insights Into Microbial Ecology (QIIME) with a cut-off of 1% and closed-reference picking (26), and three used Mothur with a dissimilarity cut-off of 3% (23, 27, 29). Three of the studies (23, 26, 27) used the Ribosomal Database Project (RDP) database, while a fourth used the Silva v.119 database (29). A single study (30) performed metaproteomic analysis using identified bacterial proteins with hierarchical clustering analysis of orthologous groups to differentiate MDD from controls, followed by phylogenetic analysis of the bacterial peptides and, with respect to Bacteroidetes and Firmicutes only, taxonomic distribution into genus and species (see Table 2 for more details).

##### Diversity assessments

All four studies assessing α-diversity (within-sample diversity) utilized the Simpson diversity index (23, 26, 27, 29), and all but one (26) also used the Shannon index. Two studies (27, 29) additionally estimated richness with abundance-based estimators, the Abundance-based Coverage Estimator (ACE) and Chao1. Generic measures for evenness (27), observed species richness (23, 26) and phylogenetic diversity (23) were also mentioned.

Estimates of β-diversity (diversity of microbial community structure) employed included weighted Bray-Curtis similarity (23), and principal coordinate analysis of weighted (29) and unweighted (23, 27) UniFrac distances.

##### Statistical analysis

To distinguish MDD from control groups, a variety of techniques was used, ranging from classical statistics to machine learning approaches. These included the following: Student's t- and Wilcoxon's signed-rank Tests (29), Mann–Whitney tests (28, 30), the linear discriminant analysis effect size method (27), principal component analysis (PCA) (26), and the Random Forest algorithm (23).

##### Functional analyses

Only the most recent investigation (30) employed methodology allowing a functional assessment of the gut microbiome. They directly measured fecal protein levels by performing high performance liquid chromatography followed by mass spectrometry, identifying non-redundant proteins on the basis of two unique peptides and filtering out human proteins. The resulting proteins were classified using the Cluster of Orthologous Groups database (138,458 proteins from 66 genomes that have been classified into 23 functional categories) in order to predict function in the gut microbiome.

#### Synthesized Findings (see Table 3)

Combining these studies, 50 taxa were significantly (*p* < 0.05) different between MDD and controls. On the phylum level, these six studies identified the following phyla as being significantly different between the two groups: *Bacteroidetes, Firmicutes, Actinobacteria, Fusobacteria*, and *Protobacteria*. Phylum *Firmicutes* had the largest number of taxa (nine families) found to be significantly different between MDD and controls. On the family level, *Lachnospiracea* was found to be significantly different between the two groups in four studies, however, two were higher and two lower in direction. All studies identified taxa at the genus level, finding 21 genera that distinguished the diagnostic groups, as follows: nine genera were higher in MDD (*Anaerostipes, Blautia, Clostridium, Klebsiella, Lachnospiraceae incertae sedis, Parabacteroides, Parasuterella, Phascolarctobacterium*, and *Streptococcus*), six were lower (*Bifidobacterium, Dialister, Escherichia/Shigella, Faecalibacterium*, and *Ruminococcus*), and six had findings in both directions (*Alistipes, Bacteroides, Megamonas, Oscillibacter, Prevotella*, and *Roseburia*). Genera identified by more than one report as elevated in MDD were *Alistipes, Blautia, Oscillibacter* and *Clostridium*, although *Alistipes* and *Oscillibacter* also had one report each of being lower. Only *Faecalibacterium* had two reports of being lower in MDD.

**Table 3:** Taxa with disparate representation in depressed vs. non-depressed humans in six case-controlled studies quantifying gut microbiome from feces.

**Phylum**	**Class**	**Order**	**Family**	**Genus**
*Actinobacteria* ↑Chen ↓Jiang	*Coriobacteridae*	*Coriobacteriales* ↑Zheng	*Coriobacteriaceae* ↑Zheng	
		*Bifidonbacteriales*	*Bifidobacteriaceae* ↑Chen	*Bifidobacterium* ↓Aizawa
		*Actinomycetales*	*Streptomycetaceae* ↑Chen	
			*Actinomycetaceae* ↑Chen	
			*Nocardiaceae* ↑Chen	
*Bacteroidetes* ↑Jiang ↑ Naseribafrouei ↓Chen ↓Lin	*Bacteroidia*	*Bacteroidales*	*Rikenellaceae* ↑Jiang ↔Chen	*Alistipes* ↑Jiang ↑Naseribafrouei ↓Zheng
			*Prevotellaceae* ↓Jiang (A-MDD) ↓Chen	*Prevotella* ↑Lin ↓Jiang
			*Bacteroidaceae* ↑Jiang (R-MDD) ↓Jiang (A-MDD)	*Bacteroides* ↑Jiang (R-MDD) ↓Jiang (A-MDD)
			*Porphyromonadaceae* ↑Jiang ↔Chen	*Parabacteroides* ↑Jiang
	*Sphingobacteria*	*Sphingobacteriales*	*Chitinophagaceae* ↓Chen	
			*Marniabilaceae* ↓Chen	
*Firmicutes* ↑Chen ↓Jiang ↑Lin	*Clostridia*	*Clostridiales*↑Zheng	*Lachnospiraceae* ↑Chen ↑Zheng ↓Jiang ↓Naseribafrouei	*Lachnospiraceae incertae sedis* ↑Jiang (A-MDD)
				*Anaerostipes* ↑Zheng
				*Blautia* ↑Jiang (A-MDD) ↑Zheng
				*Roseburia* ↑Jiang ↓Zheng
			*Oscillospiraceae* ↓Chen	*Oscillibacter* ↑Jiang (A-MDD) ↓Jiang (R-MDD) ↑Naseribafrouei
			*Ruminococcaceae* ↑Chen ↑Zheng ↓Jiang (R-MDD)	*Faecalibacterium* ↓Jiang ↓Zheng
				*Ruminococcus* ↓Jiang
			*Clostridiaceae* ↑Chen	*Clostridium* (XIX+IX) ↑Jiang (A-MDD)
				↑Lin
			Unclassified ↑Zheng	
	*Negativicutes*	*Selenomonadales*	*Veillonellaceae* ↓Jiang	*Megamonas* ↑Jiang (A-MDD) ↓Zheng
				*Dialister* ↓Jiang
			*Acidaminococcaceae* ↑Jiang ↓Zheng	*Phascolarctobacterium* ↑Jiang
	*Erysipelotrichia*	*Eerysipelotrichales*	*Erysipelotrichaceae* ↑Chen ↓Jiang (A-MDD) ↑Zheng	
	*Bacilli*	*Lactobacillus*	*Streptococcaceae*	*Streptococcus* ↑Lin
*Fusobacteria* ↑Jiang (A-MDD) ↓Jiang (R-MDD)	*Fusobacteriia*	*Fusobacteriales*	*Fusobacteriaceae* ↑Jiang (A-MDD)	
*Proteobacteria* ↑Jiang (A-MDD) ↓Chen ↓Jiang (R-MDD)	*Gammaproteobacteria*	*Enterobacteriales*	*Enterobacteriaceae* ↑Jiang ↓Chen	*Escherichia*/*Shigella* ↓Jiang (R-MDD)
				*Klebsiella* ↑Lin
	*Betaproteobacteria*	*Burkholderiales*	*Sutterellaceae* ↓Chen	*Parasutterella* ↑Jiang (A-MDD)

*Only taxa whose differences reached statistical significance (p ≤ 0.05) were selected*.

*↑, increased in depression; ↓, decreased in depression; ↔, direction not deciphered; A-MDD, Active-MDD; R-MDD, recovering-MDD exhibited 50% reduction in Hamilton Depression Score after treatment. If “Jiang” is mentioned without identifying A-MDD or R-MDD, both exhibited the same directionality*.

Results differed between the four studies that compared α-diversity between groups (23, 26, 27). One study (27) reported statistically significant higher α-diversity with respect to one (Simpson's index) of multiple estimators used, in MDD who did not respond to treatment, but not in the group of treatment-responsive MDD, compared with controls (5.344 in non-responders vs. 5.038 in controls). A second study (26) found no differences between MDD and controls with respect to species richness (374 ± 56 vs. 351 ±42, respectively) or Simpson's index for α-diversity (39.5 ± 15.9 vs 34.4 ± 19.6); however, their control group was not healthy volunteers but rather patients with diffuse neurological complaints. The third study (23) found no differences in α-diversity, while the final study described α-diversity measures in the Methods section but did not report any findings (29).

Three studies analyzed and compared β-diversity. One (27) was not able to obtain an estimate of phylogenetic β-diversity due to large inter-individual variability. Two studies reported a difference between MDD and controls with respect to β-diversity: one (23) using unweighted UniFrac analysis (eigenvalues 19, 7.2, 5.5%), and the other (29) using weighted UniFrac analysis (eigenvalues 8.6 and 6.8%).

According to the Cluster of Orthologous Group classifications, Chen et al. (30) found the four most striking group differences in fecal bacterial protein levels in the categories of translation, ribosomal structure and biogenesis; carbohydrate transport and metabolism; energy production and conversion; and inorganic ion transport and metabolism (in descending order of magnitude).

## Discussion

### Main Findings

All studies reviewed found significant differences in taxa between MDD and control groups, and one study additionally demonstrated that fecal transplantation from MDD patients into mice induced depressive-like behaviors (23). However, there was minimal consensus with regard to either microbial diversity or relative abundance or directionality of differences in taxa associated with MDD.

### Sources of Bias

#### Methodologic Differences

Likely accounting for some of the discordance among studies is discrepant methodology (see Table 2). The use of metaproteomics and phylogenetic analysis of bacterial peptides in one study (30) affects comparability with the other studies, which used 16S RNA quantification. Furthermore, in contrast to most of the studies, two articles limited their search of the gut microbiome *a priori* to specific taxa: Firmicutes and Bacteroidetes (30) or Bifidobacterium and Lactobacillus (28). Even though most studies used high throughput sequencing of 16S rRNA, the analysis methods involved different variable regions, different pipelines, different databases, and different cut-offs, which may each influence results to varying degrees. Moreover, analytic methods differed widely among the studies. Statistical methodology for microbiome analysis has not been standardized across the field, and many approaches have been noted to be prone to high false discovery rates (31), a concern addressed specifically by only two studies (26, 30).

#### Sample Differences

Although strong population homogeneity was a within-study strength, different population characteristics across studies contribute to difficulties in comparability. For example, geographic/ethnic dietary differences (32), as well as differences in host genetics, immune characteristics, and behavioral factors within each geographic population may be expected to affect the gut microbiome directly. Differences in microbial composition vary considerably by geographical location (33). In fact, regional differences within a single province in China were found to be the strongest phenotypic determinant of microbiome variability in a large sample (*N* = 7,009) of similar ancestry (99% Han Chinese), accounting for five-fold more variance than the next most relevant of the 30 attributes studied (34). Thus, a signal associated with depression would need to account for enough variance to be detected in the context of all these other effects. Of note, a study of regionally heterogeneous participants did find a signal for mental illness status that was reproducible among subsets by region, although the mental illness status was determined by self-report and covered several conditions in addition to depression (33).

Small sample sizes, as low as only 10 individuals per diagnostic group in two studies (29, 30), limit confidence in the results. Additionally, exclusion criteria varied among the six studies with respect to medical conditions. This may have resulted in heterogeneity within the control groups, as systemic inflammation due to medical conditions could be associated with differences in the microbiota between individuals.

The use of medications in all the studies included in this review is likely to also have had an impact on the gut microbiome. In particular, there is evidence in the literature that antipsychotics may impact the microbiome. For example, atypical antipsychotics are associated with altered gut microbiota in rodents (35–37). In a study of bipolar adults (38), significant changes in the abundance of three genera were identified between those taking vs. not taking antipsychotics. The inclusion of individuals on antipsychotics in two of the six studies (27, 30), therefore, contribute to the problems inherent in comparing these studies. In addition, one of the studies included individuals using probiotics (28), which may influence the composition of the gut microbiome, improve gut wall function, and influence immune system signaling (39, 40).

### Mechanisms Through Which the Microbiota may be Associated With Psychopathology

Like the six articles reviewed here, many studies of the microbiome have focused on the relative abundance of specific microbial operational taxonomic units (OTUs) and health outcomes of interest. However, looking for specific taxa as a marker of disease may miss important information for certain conditions. Although a great number of microbial taxa are found in the human gut microbiome, a limited number will take up niches in any one individual. For example, over 1,000 bacterial species were identified in a sample of 124 subjects. Each individual's feces harbored at least 160 species, but much of the bacterial DNA coded for similar processes (41). Gut microbiome distribution across individuals demonstrates surprisingly low convergence even between individuals on identical diets (42), in accord with evidence of conserved metabolic processes across taxa (43). This suggests that different individuals can have taxonomically varied but functionally similar microbiota, i.e., the same or similar essential functions can be performed by a variety of microbial taxa. Likewise, varied permutations of microbial communities can take up nutritional and locational niches depending on opportunity and environment (6). With respect to exploration of the gut microbiome's effects on depression, understanding microbial functions may therefore be more illuminating than focusing on relative abundance of specific taxa. Chen et al. (30), the most recent of the reviewed articles, attempted to address this by employing a proteomic analysis method to produce comparative functional assessment based on the identified bacterial proteins. The impact of this article is necessarily limited by the problem of generalizing from a very small sample size (*n* = 10 MDD/10 controls).

Fundamental bacterial functions involve consumption of substrates and production of metabolites (44). A taxon's specific pattern of substrate and nutrient usage may shed light on its survival fitness in certain environments and its ability to use available materials for products, some of which may be relevant to depression. Bacteria often demonstrate symbiotic relationships with other taxa, metabolizing substrates into products which may benefit themselves, feed or otherwise benefit their neighbors and host, or influence/signal their neighbors and host (44). Of particular interest with respect to depression etiology are products that can interact with the nervous systems (central and enteral) or play a role in immune responses such as inflammation. Gut microbiota benefit the host in multiple ways, include digesting/fermenting carbohydrates, producing micronutrients, mounting immune responses to discourage colonization by pathogens (45), and producing a variety of neuroactive molecules (46). Thus, multiple possible pathways exist whereby the microbiome may either contribute to or confer resilience against depression. Some instances are explored below.

#### Bacterial Metabolism of Carbohydrates and Proteins

Dietary carbohydrates, including indigestible oligosaccharides, are a common substrate for gut bacteria, which transform them into short chain fatty acids (SCFAs), including acetate, propionate, butyrate, and valerate. These substances serve as energy sources for the host and for other bacterial species (10, 47). SCFAs also trigger differentiation of T cells (48) and can function as histone deacetylase inhibitors, which as a class have immunosuppressive (49) and anti-inflammatory (50–52) functions and have been proposed as potential novel antidepressants (53). Additionally SCFAs activate G protein-coupled receptors (49), are involved in neurotransmitter production (54) and neuroprotection (55, 56), and can themselves penetrate the blood-brain barrier (57). We looked broadly at reports of SCFA production among the reviewed case-control studies and found that in general the genera *reduced* in MDD have extensive capacity to metabolize carbohydrates, particularly mono- and disaccharides and their derivatives [*Bifidobacterium* (58–62), *Faecalibacterium* (63), and *Ruminococcus* (64–66)]. *Bacteroides*, found to be reduced in a treatment-nonresponsive depressed subgroup compared to healthy controls (27), as a genus has a particularly rich armamentarium for metabolizing more complex carbohydrates including glycans of human mucin (67–78).

In contrast, although some of the genera reported as *elevated* in MDD also can metabolize carbohydrates [*Anaerostipes* (79), *Blautia* (80), *Clostridium* (65, 81, 82)] it may be noteworthy that several are high metabolizers of amino acids and proteins [*Clostridium* (83, 84), *Klebsiella* (84), *Parabacteroides* (85), *Streptococcus* (83), *Oscillibacter* and *Alistipes* (84)]. Increased metabolism of proteins by microbiota involves fermentation, or bacterial putrefaction, a process that may divert essential host amino acids from the host to the microbes and may result in toxic products such as ammonia, putrescine, and phenol (84). Dysbiosis that results in increased putrefaction has been implicated in the pathogenesis of colorectal cancer (84) and autism spectrum disorders (86). With regard to depression, in a large epidemiologic study, elevated dietary intake of protein at baseline was associated with more severe depressive symptoms at 10-year follow-up in a dose-dependent manner, in women only (87). Others have reported that interactions between dietary protein levels and genetic polymorphisms can moderate the risk of depression (88).

Thus, while not quantitatively testable across the studies reviewed here, we may speculate that dysbiosis resulting in a relatively lower capacity to metabolize carbohydrates and higher capacity for protein metabolism may have a role in the pathogenesis of MDD. Lower SCFA could contribute to symptomatology as a result of lower energy and altered neurotransmission, while both lower SCFA and higher putrefaction products are implicated in intestinal inflammation, relevant here as inflammatory bowel conditions have been associated with a high co-morbidity with depression and anxiety (89). We have focused our discussion on mechanisms through which dysbiosis could contribute to the development of depression. However, the directionality of the associations between inflammatory bowel disease and depression is not known. Thus, it is also plausible that depression may cause digestive problems and dysbiosis either through altered dietary choices or other mechanisms. One hypothesis could be that in some individuals, depression confers a decreased ability to digest proteins; the resulting increased residual protein in the colon would give an advantage to higher concentrations of microbes which prefer proteins as substrates, leading to higher putrefaction and inflammation.

#### Production of Micronutrients

Water-soluble vitamins from the diet may be absorbed from the small intestine, including ascorbate (vitamin C), biotin (B7), folate (B9), niacin (B3), pantothenic acid (B5) pyridoxine (B6), riboflavin (B2), and thiamine (B1) (90). However, a number of these water-soluble vitamins also can be generated by microbiota and absorbed in the colon and thereby affect the host (90). Therefore, dysbiosis resulting in low production of micronutrients could contribute to depression pathophysiology, particularly in the case of depressed patients with poor nutritional intake. For example, *Bifidobacterium*, among the genera that were less abundant in MDD in reviewed case-control studies, can synthesize riboflavin, niacin, and folate (91). Low folate levels have been associated with the presence of depression by meta-analysis (92), and folate levels were inversely associated with depressive symptom severity in one epidemiological study (93). The relative contribution of dietary *vs*. bacterial folate to the host is not clear (90); however, a carrier for uptake of folate into human colonocytes has been described in cell culture (94) and *ex vivo* (95) studies. Deficiency of thiamine is implicated in depressive symptoms in older adults (96). Microbial production results in a pool of free, absorbable thiamine and, similarly to folate, specific carrier proteins exist to transport free thiamine into both intestinal enterocytes and colonocytes (97). Microbial production of vitamins also may affect humans indirectly in a kind of food chain where vitamins produced by certain microbes are needed by other microbiota whose downstream products impact depression. For example, about half of human gut microbes are thiamine auxotrophs, i.e., they require but cannot make their own thiamine (98).

#### Inflammatory Regulation

Depression and anxiety symptoms increase with functional gut disorders (99). Irritable bowel syndrome (IBS) has been characterized by increased permeability of the mucosal layer (100, 101) as well as gut microbiome dysbiosis (102). Inflammation can compromise normal barriers protecting the body from pathogenic gut bacteria (8, 103), resulting in intestinal permeability (104), or “leaky gut,” and even leakage at the blood-brain barrier (105, 106). One mechanism whereby this occurs is tumor necrosis factor alpha (TNFα) induction of shedding and apopotosis of intestinal epithelial cells (107). This has been suggested as an explanation for the high association of inflammatory gastrointestinal disorders with depression; for example, 49% of people with inflammatory bowel disease suffer from depressive symptoms (89). Gut dysbiosis promotion of inflammation may contribute to multiple pathways in the CNS that are implicated in the development of depression. Upregulation of inflammatory cytokines (108) has downstream consequences in brain, including shunting of tryptophan away from serotonin synthesis toward the kynurenine pathway (109) as well as excitotoxic and neurotoxic effects (110, 111).

#### Production of Neurotransmitters

In cell culture studies, gut microbiota have been found to make precursers to neurotransmitters, such as tryptamine (112), and neurotransmitters including GABA (9), serotonin, norepinephrine, and dopamine (57). Among the bacteria found in the reviewed studies to be lower in MDD (28), *Bifidobacterium* is an efficient producer of GABA (113). Several bacterial strains are known to produce serotonin directly (114). Moreover, one study reported that male rodents reared in a germ-free environment, a profound manipulation of the microbiome, exhibit increased levels of serotonin in the hippocampus, along with increased peripheral levels of tryptophan, a serotonin precursor, suggesting the possibility of a peripheral origin to this effect (21). Another animal study found that administration of a probiotic containing *Lactobacillus plantarum* PS128 led to both antidepressant-like effects in mice as well as increases in levels of serotonin and dopamine in the striatum (115). Modulation of neurotransmitter production is one possible means by which the gut microbiome may affect the brain, with direct relevance to depression.

### Human Brain Imaging Supporting Gut Microbiome-Brain Communication

The current literature supports bidirectional interactions between the gut and brain mediated by gut microbiota (25, 116, 117). There is evidence that gut microbiome composition is correlated with neural activity and brain structure in humans, as assessed by functional and structural MRI (118, 119). Specificially, an observational study in obese and non-obese individuals found that both microbial diversity as well as relative abundance of *Actinobacteria* were associated with measures of white matter integrity and of regional iron content in the brain (118). Another study in healthy female volunteers identified two clusters of individuals based on bacterial genotyping, a *Bacteroides*-abundant group and a *Prevotella*-abundant group, and observed differences in measures of both structural and functional neuroimaging when comparing these groups (119). Both *Bacteroides* (27) and *Prevotella* (27, 29) were associated with MDD in specific case-control studies and exhibited divergent directionality.

Moreover, probiotic interventions can alter neural responses assessed by fMRI (120, 121). Specifically, randomized, double-blind, placebo-controlled treatment with *Bifidobacterium longum* in patients with irritable bowel syndrome led to reduction in neural responses to emotionally negative stimuli in limbic brain regions including the amygdala (121). Likewise, *B. longum* administration to mice via oral gavage had an anxiolytic effect, and this was not seen in vagotomized animals (116). Another small randomized trial in healthy women found that administration of a probiotic containing multiple species was associated with a decrease in neural responses to an emotional faces attention task in a broad network of brain regions, including insula and somatosensory processing regions (120). In rodents, oral gavage administration of *Lactobacillus rhamnosus*, which improved anxiety- and depression-like behaviors, also caused brain region-specific alterations in GABA_Aα2_ mRNA expression detected by *in situ* hybridization (117). Neither the neurochemical nor the behavioral effects were seen in vagotomized animals, indicating that the vagus nerve mediated this particular communication between the gut microbiota and brain.

Important features of gut-brain communication that require further elucidation with respect to influences in MDD include the different roles of luminal vs. systemic bacteria, the mechanisms of traversing the gut barrier into the portal circulation and the blood-brain barrier (BBB), and the role of the vagus nerve.

### The Role of Diet

Dietary effects on the gut microbiome are evident as early as infancy, with gut microbiome differences observed between breast-fed and formula-fed infants, including a greater prevalence of *Bifidobacteria* in breast-fed infants (122). In adulthood, gut microbiome composition is associated with self-reported long-term dietary patterns, with a higher prevalence of *Prevotella* observed in individuals reporting higher intake of carbohydrates and simple sugars, and a higher prevalence of *Bacteroides* observed in individuals reporting higher intake of animal protein and saturated fats (32). Effects of short-term dietary interventions on the gut microbiome are not well understood. As one example, probiotic administration in a recent study led not only to changes in brain activity assessed by fMRI (see section Human Brain Imaging Supporting Gut Microbiome-Brain Communication above), but also to antidepressant effects and changes in urine metabolic profiles, suggestive of microbiome effects, but no measureable effects on gut microbiome composition were detected by fecal 16S rRNA gene sequencing (121). It is unclear to what extent this finding reflects limitations in our current microbiome quantification tools, or resistance of the gut microbiome to change.

One dietary factor in the microbiome-inflammation-depression relationship is the intake of polyunsaturated fatty acids (PUFAs), especially with regard to the ratio of omega-3 to omega-6 PUFAs, which has been found to be low in depression (123). Omega-3 PUFAs tend to be anti-inflammatory while omega-6 PUFAs such as arachidonic acid tend to be pro-inflammatory [reviewed in (124)], and the PUFA balance has effects on mucus adhesion of bacteria (125). For example, in a study of gnotobiotic piglets the growth and mucus adhesion of probiotic *Lactobacillus paracasei* were enhanced by concomitant administration of a mixture of PUFAs (126). Omega-6 PUFAs also are implicated in epithelial permeability and mucosal damage through the generation of leukotrienes, inflammatory metabolites of arachidonic acid (127). Dietary PUFA serves as a substrate for some gut microbes, and PUFA-derived metabolites have been proposed as novel gut microbial products that may have important physiological effects (16, 128). Among the genera reported in the reviewed case-control studies as less abundant in MDD, *Bifidobacterium* degrades unsaturated fatty acids including linoleic acid (129), a precursor to arachidonic acid.

### Current Analytic Tools

The analysis of trillions of gut microorganisms with respect to genomic composition and functionality, which includes a complex ecological network of interactions among bacteria and with the host, is truly a “big data” enterprise. Increasingly sophisticated systems biology techniques are coming on line to perform these analyses, among them genome-scale metabolic modeling (GEM), which aims to comprehensively describe the metabolism of a microbe or a microbial community (130). These systems were conceptualized 30 years ago and are now reaching the stage of development where they can begin to be utilized for predictive biology approaches (131). For example, the GEM for *Escherichia coli* utilizes existing information of 66% of its genome and has enabled a wide variety of applications including models for interspecies metabolite exchange (132). Relatively new analysis methods include shotgun metagenomic sequencing, a powerful approach from which coding sequences can be annotated with respect to known metabolic pathways, allowing for functional inferences. However, this method also has drawbacks, including expense, biased representation of genome catalogs and functional annotations, sequencing of dead cells, distortions of relative abundance reporting, and minimal success in regard to mucosal microbiota (133). Another approach utilizes PICRUSt (Phylogenetic Investigation of Communities by Reconstruction of Unobserved States), a bioinformatic tool that allows for estimating the functional gene profile of a metagenome using marker gene data (i.e., 16S sequences) and a set of known genomes. This tool applies an ancestral state reconstruction algorithm that predicts the presence of gene families and then combines these into a metagenome. As such it provides a predictive metagenome and offers insights into functional capacities of uncultured bacterial communities. Other novel indices may also have greater merit for distinguishing between affected and unaffected populations than abundance or diversity measurements. For example, the growth rate of the bacterial population, assessed as the ratio of DNA copy number of DNA regions near the replication origin to those near the terminus, has been shown to correlate with illness better than species abundance in inflammatory bowel disease and type II diabetes (134).

### Developing Standards for Future Studies

Major sources of controllable clinical heterogeneity identified in this review that can affect quantitative comparability of clinical trials include: (1) psychiatric medication status; (2) type of comparison group; (3) medical comorbidities; (4) non-antibiotic medications impacting the gut microbiome (135); (5) psychiatric comorbidities including substance use disorders; and (6) use of well-validated diagnostic and depression severity assessment instruments.

Although bioinformatic analysis pipelines in general are well-standardized, handling of specimens, DNA extraction protocols and sequencing methods introduce another important type of heterogeneity.

Geographic differences may have a profound impact on study outcomes (32–34), reflecting differences in ethnicity, environmental exposures, or diet. Attempts to control statistically for location found interpolation to be more successful than an extrapolative approach (34).

The use of a standard panel of covariates would be an essential approach to reduce the “noise” within which the depression signal will need to be detected. Standards should include assessment of diet, although this is difficult to achieve comprehensively. One aspect of diet that may be important to quantify with respect to depression includes consumption of wine, a major source of polyphenols that interact with the microbiome resulting in decreased fecal concentrations of inflammatory cytokines (136). Easily quantified non-dietary information that has a robust effect size on the gut microbiome has been reported in studies (137, 138) using data from the Belgian Flemish Gut Flora Project (FGFP; *N* = 1,106) and the Dutch LifeLines-DEEP study (LLDeep; *N* = 1,135), as well a larger study from China (*N* = 7,009) (34). Considering non-dietary covariates, all three studies found Bristol stool score, age, sex, body-mass index, triglycerides, cholesterol, and smoking status to be among the characteristics most strongly associated with microbiome composition.

This demonstrated need to build statistical models that control for multiple covariates mandates larger sample sizes. Without more studies, effect sizes for depression association with the microbiome are presently uncertain. However, several approaches may increase power to detect a role of dysbiosis in mood disorders. One strategy is to recruit individuals with extreme illness phenotypes. This has been used successfully in psychiatric (139) and non-psychiatric (140) medical research to elucidate pathophysiology. Applying this principle to the case of mood disorders, studies of individuals with recurrent depressive episodes, history of suicidality, or current inpatient treatment may represent a more extreme phenotype with greater likelihood of demonstrating biological abnormalities including dysbiosis. Given the significant clinical and biological heterogeneity of depression, focusing on a more narrow, severe phenotype may increase statistical power in microbiome studies (141).

Case-control designs are limited by the potential for confounding factors between patient and control groups. Aspects of study design that may further increase power of future microbiome studies of depression include dense clinical phenotyping of depression severity and symptom profiles using well-validated assessment tools including the HAM (142) and the Beck Depression Inventory (143). This would allow for within-group correlation analyses, as well as longitudinal microbiome quantification within patient groups, harnessing the statistical power of within-subject statistical designs to examine questions of state vs. trait abnormalities.

Thus, ideal study conditions would feature a large sample of more severely ill, unmedicated patients diagnosed with MDD and assessed for severity by trained clinical researchers using validated instruments, in comparison to comprehensively assessed healthy volunteers matched for sex, age, race, and geographic/ethnic background. Subjects with current substance use disorders or active medical illness, including chronic inflammatory conditions, would be excluded from both MDD and psychiatrically healthy volunteer groups. Comprehensive diagnostic information obtained would include details on past substance use history. Subjects would complete dietary assessments, such as the USFDA's Healthy Eating Index (144), which has been validated for studying the microbiome (145), and this information plus data on Bristol stool score, body-mass index, triglycerides, cholesterol, and smoking status would be included as covariates in statistical modeling. Interpretation of the results would need to include rigorous control for false discovery rates.

Similar to the situation with depression, the study of the microbiome across a range of other neuropsychiatric conditions is in its infancy (146), e.g., reviews of human research on dysbiosis report on only 12 articles concerning autism (147) and five articles concerning schizophrenia and bipolar disorder combined (148). When greater consensus is reached regarding the nature of dysbiosis in individual neuropsychiatric conditions, perhaps by employing techniques advocated here for standardization and sample selection, an important question will be to determine the degree of overlap in findings across these conditions. The use of an open science approach, creating data sharing and data repositories specific to psychiatric illness, will be important to advancing the field of what may be called the psychomicrobiome. Such an approach would facilitate amassing sample sizes that are sufficiently powered to detect the effects of the confounding variables described above and to detect what may be modest effect sizes in primary contrasts of interest.

### Limitations

Our review of this literature is limited to a descriptive approach. Because these studies were disparate in their aims and methodologies, studied heterogeneous populations, reported on relative rather than absolute abundance, and were small and likely underpowered, a meta-analytic approach would be handicapped.

The search for gut microbiota via stool carries specific limitations. Location of the taxa within the gut may be an important determinant of microbial effects on the host organism (46), but stool may represent luminal and lower intestinal populations, and composition differs between upper and lower intestine and between luminal and mucosal layers (149). Additionally, it may not be clear whether a taxon maintains a stable population within the host (autochthonous) or is just passing through (allochthonous); feces may consist of allochthonous taxa plus some particularly proliferative and not so adherent authochthonous taxa (6). We further note that other sites of the human microbiome may be equally or more relevant but are understudied with regard to depression, e.g., the oral cavity [already used in human studies of anxiety (150), stress (151), autism spectrum disorders (152), and migraine (153)], which can be easily and directly sampled.

## Conclusions

The limited existing human studies of depression and gut microbiota report depression-specific findings regarding proportions of microbiota; however, the sample sizes are small, and no consensus has emerged concerning which bacterial taxa are most relevant to depression. Therefore, the effect size of depression-related microbial differences is presently unclear. Future, larger studies may employ a functional system of categorization, and should evaluate BMI, presence of inflammation, and diet, and control for other factors that may be important covariates. Studying depressed patients who are unmedicated at the time of microbiome assessment and in an active depressed state may help address possible confounds of antidepressant medication effects and state vs. trait effects. Improved resolution at the species level may be achieved using next-generation sequencing approaches. Given the evidence of microbial function that transcends taxonomic classification, and the ecological nature of the microbial community, approaches such as bacterial growth rate and genome-scale metabolic modeling may prove useful in better understanding the contribution of the gut microbiome to depression etiology.

## Author Contributions

JMM, MES, AG, and SC conceptualized the project. AG and SC performed the literature review. SC wrote the first draft of the manuscript. JJM and A-CU critically reviewed the manuscript with respect to their areas of expertise: depression (JJM) and gut microbiology (A-CU). All authors contributed to writing and revising the manuscript.

### Conflict of Interest Statement

JJM receives royalties for the commercial use of the C-SSRS from the Research Foundation for Mental Hygiene. A-CU received research funding from Allergan and GSK unrelated to the current study. The funders had no role in study design, data collection and analysis, decision to publish, or preparation of the manuscript. The remaining authors declare that the research was conducted in the absence of any commercial or financial relationships that could be construed as a potential conflict of interest.
